# Supplementary material for: MRI Radiomics Data Analysis for Differentiation between Malignant Mixed Müllerian Tumors and Endometrial Carcinoma
Source: Cancers (Basel). 2024 Jul 25;16(15):2647. doi: 10.3390/cancers16152647 (PMC11312193; doi:10.3390/cancers16152647)
Supplement: Supplementary file 1 [file cancers-16-02647-s001.zip › cancers-3094381-supplementary.pdf]

First-Order Statistics:
- Average measures
- Range measures

Angular Second-Order Statistics:

- Angular variance measures

Different Scale Levels:

- 8x8 window
- 16x16 window
- 32x32 window
- 64x64 window
- 256x256 window.

Subvariables:

- Autocorrelation,
- Contrast
- Correlation
- Cluster Prominence
- Cluster Shade
- Dissimilarity
- Energy
- Entropy
- Homogeneity
- Maximum Probability
- Sum of Squares: Variance
- Sum Average,
- Sum Variance
- Sum Entropy
- Difference Variance

- Difference Entropy
- Information Measure of Correlation 1
- Information Measure of Correlation 2
- Inverse Difference Normalized (INN)
- Inverse Difference Moment Normalized.

Texture features are often categorized into groups based on the underlying texture properties they capture.

First-Order Statistics:

- Average of Autocorrelation
- Average of Contrast
- Average of Correlation
- Average of Cluster Prominence
- Average of Cluster Shade
- Average of Dissimilarity
- Average of Energy
- Average of Entropy
- Average of Homogeneity
- Average of Maximum Probability
- Average of Sum of Squares: Variance
- Average of Sum Average
- Average of Sum Variance
- Average of Sum Entropy
- Average of Difference Variance
- Average of Difference Entropy
- Average of Information Measure of Correlation 1 Average of Information Measure of Correlation 2
- Average of Inverse Difference Normalized (INN) Average of Inverse Difference Moment Normalized

Range Measures:

- Range of Autocorrelation
- Range of Contrast

- Range of Correlation
- Range of Cluster Prominence
- Range of Cluster Shade
- Range of Dissimilarity
- Range of Energy
- Range of Entropy
- Range of Homogeneity
- Range of Maximum Probability
- Range of Sum of Squares: Variance
- Range of Sum Average
- Range of Sum Variance
- Range of Sum Entropy Range of Difference Variance
- Range of Difference Entropy
- Range of Information Measure of Correlation 1
- Range of Information Measure of Correlation 2
- Range of Inverse Difference Normalized (INN) Range of Inverse Difference Moment Normalized

Angular Second-Order Statistics:

- Angular Variance of Autocorrelation
- Angular Variance of Contrast
- Angular Variance of Correlation
- Angular Variance of Cluster Prominence
- Angular Variance of Cluster Shade
- Angular Variance of Dissimilarity
- Angular Variance of Energy
- Angular Variance of Entropy
- Angular Variance of Homogeneity
- Angular Variance of Maximum Probability
- Angular Variance of Sum of Squares: Variance
- Angular Variance of Sum Average
- Angular Variance of Sum Variance

- Angular Variance of Sum Entropy
- Angular Variance of Difference Variance
- Angular Variance of Difference Entropy
- Angular Variance of Information Measure of Correlation 1
- Angular Variance of Information Measure of Correlation 2 Angular Variance of Inverse Difference Normalized (INN)
- Angular Variance of Inverse Difference Moment Normalized.

Supplement Table S1. Univariate analysis AUC ROC and ordered by AUC

Predictor	N	AUC	LL	UL	p-value
Volume	61	0.7056	0.5692	0.8419	0.00618
..32Range.of.Cluster.Shade	61	0.6989	0.5576	0.8402	0.00815
..256Angular.Variance.of.Cluster.Shade	61	0.6989	0.5569	0.8409	0.00815
..256Range.of.Cluster.Shade	61	0.6978	0.5559	0.8396	0.00853
..64Range.of.Cluster.Shade	61	0.6967	0.5548	0.8385	0.00893
..64Angular.Variance.of.Cluster.Shade	61	0.6967	0.5542	0.8391	0.00893
..32Angular.Variance.of.Cluster.Shade	61	0.6944	0.5517	0.8372	0.00976
..16Angular.Variance.of.Cluster.Shade	61	0.69	0.5472	0.8328	0.0116
..16Range.of.Cluster.Shade	61	0.6856	0.5424	0.8288	0.0138
..8Range.of.Sum.of.squares..Variance	61	0.6811	0.5447	0.8175	0.0164
..8Angular.Variance.of.Cluster.Shade	61	0.68	0.5370	0.823	0.0171
..8Angular.Variance.of.Sum.average	61	0.68	0.5436	0.8164	0.0171
..32Range.of.Sum.average	61	0.68	0.5432	0.8168	0.0171
..8Range.of.Sum.average	61	0.6778	0.5409	0.8146	0.0185
..16Angular.Variance.of.Sum.average	61	0.6778	0.5415	0.814	0.0185
..32Angular.Variance.of.Sum.average	61	0.6767	0.5403	0.813	0.0193

..64Angular.Variance.of.Sum.average	61	0.6767	0.5401	0.8133	0.0193
..256Angular.Variance.of.Sum.average	61	0.6767	0.5401	0.8133	0.0193
..64Range.of.Sum.average	61	0.6744	0.5367	0.8121	0.0209
..256Range.of.Sum.average	61	0.6744	0.5369	0.812	0.0209
..16Range.of.Sum.average	61	0.6733	0.5362	0.8105	0.0218
..8Angular.Variance.of.Sum.of.squares..Variance	61	0.6711	0.5341	0.8081	0.0236
..16Range.of.Sum.of.squares..Variance	61	0.6689	0.5311	0.8067	0.0255
..256Range.of.Information.measure.of.correlation.1	61	0.6689	0.5282	0.8096	0.0255
..256Angular.Variance.of.Information.measure.of.correlation.1	61	0.6678	0.5263	0.8092	0.0265
..256Angular.Variance.of.Entropy	61	0.6667	0.5241	0.8092	0.0276
..16Angular.Variance.of.Sum.of.squares..Variance	61	0.6644	0.5260	0.8029	0.0298
..8Range.of.Cluster.Shade	61	0.6622	0.5182	0.8063	0.0321
..256Range.of.Entropy	61	0.6622	0.5188	0.8056	0.0321
..16Average.of.Cluster.Shade	61	0.6611	0.5240	0.7982	0.0334
..64Average.of.Cluster.Shade	61	0.6611	0.5241	0.7981	0.0334
..32Average.of.Cluster.Shade	61	0.66	0.5230	0.797	0.0346
..32Range.of.Sum.of.squares..Variance	61	0.66	0.5209	0.7991	0.0346
..64Range.of.Sum.of.squares..Variance	61	0.66	0.5212	0.7988	0.0346
..256Average.of.Cluster.Shade	61	0.66	0.5228	0.7972	0.0346
..32Angular.Variance.of.Sum.of.squares..Variance	61	0.6589	0.5198	0.798	0.036
..256Range.of.Sum.of.squares..Variance	61	0.6578	0.5186	0.7969	0.0373
..256Range.of.Information.measure.of.correlation.2	61	0.6578	0.5150	0.8006	0.0373
..256Angular.Variance.of.Information.measure.of.correlation.2	61	0.6578	0.5150	0.8005	0.0373
..8Average.of.Cluster.Shade	61	0.6567	0.5194	0.794	0.0387

..16Average.of.Cluster.Prominence	61	0.6567	0.5162	0.7972	0.0387
..64Angular.Variance.of.Sum.of.squares..Variance	61	0.6567	0.5179	0.7955	0.0387
..8Range.of.Sum.entropy	61	0.6556	0.5159	0.7952	0.0401
..8Average.of.Cluster.Prominence	61	0.6544	0.5134	0.7955	0.0416
..256Angular.Variance.of.Sum.of.squares..Variance	61	0.6533	0.5140	0.7926	0.0432
Skewness	61	0.6522	0.5133	0.7912	0.0447
..32Average.of.Cluster.Prominence	61	0.6522	0.5115	0.7929	0.0447
..64Angular.Variance.of.Information.measure.of.correlation.1	61	0.6522	0.5086	0.7959	0.0447
..64Average.of.Cluster.Prominence	61	0.65	0.5091	0.7909	0.048
..64Angular.Variance.of.Entropy	61	0.6489	0.5041	0.7936	0.0497
..256Average.of.Cluster.Prominence	61	0.6489	0.5078	0.79	0.0497
..32Average.of.Sum.average	61	0.6478	0.5043	0.7913	0.0515
..64Average.of.Sum.average	61	0.6478	0.5043	0.7913	0.0515
..256Average.of.Sum.average	61	0.6478	0.5043	0.7913	0.0515
..8Average.of.Sum.average	61	0.6467	0.5027	0.7906	0.0533
..16Average.of.Sum.average	61	0.6467	0.5031	0.7902	0.0533
..8Angular.Variance.of.Sum.entropy	61	0.6444	0.5035	0.7854	0.0571
..256Angular.Variance.of.Homogeneity	61	0.6444	0.4989	0.79	0.0571
..256Average.of.Entropy	61	0.6411	0.4934	0.7888	0.0633
..256Average.of.Maximum.probability	61	0.64	0.4930	0.787	0.0654
..256Average.of.Information.measure.of.correlation.1	61	0.64	0.4955	0.7845	0.0654
..8Average.of.Sum.variance	61	0.6344	0.4887	0.7802	0.0771
..32Angular.Variance.of.Cluster.Prominence	61	0.6333	0.4808	0.7859	0.0796
..32Range.of.Cluster.Prominence	61	0.6322	0.4790	0.7855	0.0822

..64Range.of.Cluster.Prominence	61	0.6322	0.4785	0.7859	0.0822
..64Angular.Variance.of.Cluster.Prominence	61	0.6311	0.4779	0.7843	0.0849
..256Average.of.Information.measure.of.correlation.2	61	0.6311	0.4854	0.7768	0.0849
..256Angular.Variance.of.Cluster.Prominence	61	0.6311	0.4778	0.7844	0.0849
..256Range.of.Cluster.Prominence	61	0.63	0.4764	0.7836	0.0876
..8Average.of.Autocorrelation	61	0.6289	0.4833	0.7745	0.0904
..16Average.of.Autocorrelation	61	0.6289	0.4829	0.7749	0.0904
..16Average.of.Sum.variance	61	0.6289	0.4828	0.7749	0.0904
..32Average.of.Autocorrelation	61	0.6289	0.4825	0.7752	0.0904
..64Average.of.Autocorrelation	61	0.6289	0.4825	0.7752	0.0904
..256Average.of.Autocorrelation	61	0.6289	0.4825	0.7752	0.0904
..16Range.of.Cluster.Prominence	61	0.6267	0.4728	0.7805	0.0962
..32Average.of.Sum.variance	61	0.6267	0.4803	0.773	0.0962
..64Average.of.Sum.variance	61	0.6267	0.4807	0.7727	0.0962
..256Average.of.Energy	61	0.6267	0.4783	0.7751	0.0962
..256Average.of.Sum.variance	61	0.6267	0.4807	0.7727	0.0962
..16Angular.Variance.of.Cluster.Prominence	61	0.6256	0.4723	0.7788	0.0992
..64Range.of.Information.measure.of.correlation.1	61	0.6256	0.4808	0.7704	0.0992
..64Range.of.Inverse.difference.normalized..INN.	61	0.6211	0.4662	0.7761	0.112
..256Range.of.Dissimilarity	61	0.62	0.4650	0.775	0.115
..256Range.of.Inverse.difference.normalized..INN.	61	0.6189	0.4639	0.7739	0.119
..8Average.of.Sum.of.squares..Variance	61	0.6178	0.4708	0.7648	0.122
..64Range.of.Dissimilarity	61	0.6178	0.4632	0.7724	0.122
..64Range.of.Entropy	61	0.6178	0.4711	0.7644	0.122

..256Average.of.Difference.entropy	61	0.6178	0.4577	0.7778	0.122
..16Average.of.Sum.of.squares..Variance	61	0.6167	0.4696	0.7637	0.126
..32Range.of.Inverse.difference.normalized..INN.	61	0.6167	0.4622	0.7712	0.126
..16Range.of.Inverse.difference.normalized..INN.	61	0.6156	0.4605	0.7706	0.13
..32Average.of.Sum.of.squares..Variance	61	0.6156	0.4685	0.7627	0.13
..32Range.of.Dissimilarity	61	0.6156	0.4609	0.7702	0.13
..64Average.of.Sum.of.squares..Variance	61	0.6156	0.4685	0.7627	0.13
..256Average.of.Sum.of.squares..Variance	61	0.6156	0.4685	0.7627	0.13
..16Range.of.Dissimilarity	61	0.6122	0.4571	0.7673	0.141
..32Angular.Variance.of.Difference.variance	61	0.6122	0.4487	0.7757	0.141
..64Angular.Variance.of.Difference.variance	61	0.6122	0.4488	0.7756	0.141
..8Angular.Variance.of.Cluster.Prominence	61	0.6111	0.4565	0.7657	0.145
..32Range.of.Difference.variance	61	0.6111	0.4499	0.7723	0.145
..16Range.of.Difference.variance	61	0.61	0.4486	0.7714	0.15
..8Range.of.Cluster.Prominence	61	0.6089	0.4524	0.7653	0.154
..16Angular.Variance.of.Difference.variance	61	0.6089	0.4475	0.7703	0.154
..256Average.of.Difference.variance	61	0.6089	0.4479	0.7698	0.154
..16Range.of.Difference.entropy	61	0.6078	0.4519	0.7637	0.158
..32Angular.Variance.of.Contrast	61	0.6078	0.4468	0.7687	0.158
..32Angular.Variance.of.Inverse.difference.moment.normalized	61	0.6078	0.4468	0.7688	0.158
..64Range.of.Difference.variance	61	0.6078	0.4454	0.7702	0.158
..256Range.of.Homogeneity	61	0.6078	0.4609	0.7546	0.158
..256Angular.Variance.of.Dissimilarity	61	0.6067	0.4499	0.7634	0.163
..256Angular.Variance.of.Inverse.difference.normalized..INN.	61	0.6067	0.4496	0.7638	0.163

..16Angular.Variance.of.Contrast	61	0.6056	0.4436	0.7675	0.167
..16Angular.Variance.of.Dissimilarity	61	0.6056	0.4492	0.7619	0.167
..16Angular.Variance.of.Inverse.difference.moment.normalized	61	0.6056	0.4440	0.7671	0.167
..64Angular.Variance.of.Contrast	61	0.6056	0.4439	0.7673	0.167
..64Angular.Variance.of.Inverse.difference.normalized..INN.	61	0.6056	0.4484	0.7627	0.167
..64Angular.Variance.of.Inverse.difference.moment.normalized	61	0.6056	0.4439	0.7673	0.167
..32Angular.Variance.of.Dissimilarity	61	0.6044	0.4479	0.761	0.172
..64Angular.Variance.of.Dissimilarity	61	0.6044	0.4476	0.7613	0.172
..64Average.of.Difference.variance	61	0.6033	0.4436	0.763	0.176
..64Range.of.Contrast	61	0.6033	0.4424	0.7643	0.176
..256Angular.Variance.of.Contrast	61	0.6033	0.4418	0.7648	0.176
..256Angular.Variance.of.Inverse.difference.moment.normalized	61	0.6033	0.4417	0.765	0.176
..16Range.of.Contrast	61	0.6022	0.4402	0.7642	0.181
..16Range.of.Inverse.difference.moment.normalized	61	0.6022	0.4402	0.7642	0.181
..32Range.of.Contrast	61	0.6022	0.4406	0.7638	0.181
..32Angular.Variance.of.Inverse.difference.normalized..INN.	61	0.6022	0.4462	0.7583	0.181
..64Range.of.Inverse.difference.moment.normalized	61	0.6022	0.4410	0.7634	0.181
..256Range.of.Contrast	61	0.6022	0.4413	0.7631	0.181
..256Range.of.Sum.entropy	61	0.6022	0.4482	0.7563	0.181
..32Range.of.Inverse.difference.moment.normalized	61	0.6011	0.4397	0.7625	0.186
..256Angular.Variance.of.Difference.variance	61	0.6011	0.4347	0.7675	0.186
..16Angular.Variance.of.Inverse.difference.normalized..INN.	61	0.6	0.4444	0.7556	0.191
..32Average.of.Difference.variance	61	0.6	0.4388	0.7612	0.191
..256Range.of.Difference.variance	61	0.5989	0.4329	0.7649	0.196

..256Range.of.Inverse.difference.moment.normalized	61	0.5989	0.4380	0.7598	0.196
..8Range.of.Contrast	61	0.5978	0.4360	0.7595	0.201
..8Range.of.Inverse.difference.moment.normalized	61	0.5978	0.4366	0.7589	0.201
..8Range.of.Dissimilarity	61	0.5967	0.4424	0.751	0.206
..64Angular.Variance.of.Information.measure.of.correlation.2	61	0.5967	0.4496	0.7437	0.206
..8Angular.Variance.of.Contrast	61	0.5956	0.4338	0.7573	0.212
..16Average.of.Difference.variance	61	0.5956	0.4351	0.756	0.212
..64Average.of.Contrast	61	0.5956	0.4350	0.7561	0.212
..256Average.of.Homogeneity	61	0.5956	0.4388	0.7523	0.212
..8Range.of.Difference.variance	61	0.5944	0.4336	0.7553	0.217
..8Angular.Variance.of.Inverse.difference.moment.normalized	61	0.5944	0.4327	0.7562	0.217
..32Average.of.Contrast	61	0.5933	0.4325	0.7541	0.223
..32Average.of.Inverse.difference.moment.normalized	61	0.5933	0.4327	0.754	0.223
..32Range.of.Difference.entropy	61	0.5933	0.4367	0.7499	0.223
..64Average.of.Inverse.difference.moment.normalized	61	0.5933	0.4326	0.7541	0.223
..16verage.of.Difference.entropy	61	0.5922	0.4329	0.7515	0.228
..16Average.of.Inverse.difference.moment.normalized	61	0.5922	0.4317	0.7527	0.228
..32Average.of.Difference.entropy	61	0.5922	0.4321	0.7524	0.228
..8Angular.Variance.of.Difference.variance	61	0.5911	0.4310	0.7513	0.234
..16Average.of.Contrast	61	0.5911	0.4303	0.7519	0.234
..16Angular.Variance.of.Difference.entropy	61	0.5911	0.4306	0.7516	0.234
..64Range.of.Information.measure.of.correlation.2	61	0.5911	0.4446	0.7376	0.234
..256Average.of.Contrast	61	0.5911	0.4304	0.7519	0.234
..256Average.of.Inverse.difference.moment.normalized	61	0.5911	0.4304	0.7519	0.234

..256Angular.Variance.of.Energy	61	0.5911	0.4452	0.7371	0.234
..16Average.of.Dissimilarity	61	0.59	0.4301	0.7499	0.24
..32Average.of.Homogeneity	61	0.59	0.4313	0.7487	0.24
..32Average.of.Inverse.difference.normalized..INN.	61	0.59	0.4299	0.7501	0.24
..16Average.of.Homogeneity	61	0.5889	0.4307	0.7471	0.246
..16Average.of.Inverse.difference.normalized..INN.	61	0.5889	0.4293	0.7485	0.246
..8Average.of.Difference.entropy	61	0.5878	0.4285	0.7471	0.252
..32Average.of.Dissimilarity	61	0.5878	0.4272	0.7484	0.252
..64Average.of.Homogeneity	61	0.5878	0.4292	0.7463	0.252
..8Average.of.Contrast	61	0.5867	0.4269	0.7464	0.258
..8Range.of.Entropy	61	0.5867	0.4311	0.7422	0.258
..64Average.of.Difference.entropy	61	0.5867	0.4258	0.7475	0.258
..8Angular.Variance.of.Dissimilarity	61	0.5856	0.4292	0.7419	0.264
..64Average.of.Dissimilarity	61	0.5856	0.4251	0.746	0.264
..64Average.of.Inverse.difference.normalized..INN.	61	0.5856	0.4255	0.7456	0.264
..8Average.of.Inverse.difference.moment.normalized	61	0.5844	0.4248	0.7441	0.27
..8Range.of.Inverse.difference.normalized..INN.	61	0.5844	0.4286	0.7403	0.27
..8Angular.Variance.of.Difference.entropy	61	0.5844	0.4207	0.7482	0.27
..256Average.of.Dissimilarity	61	0.5844	0.4237	0.7452	0.27
..256Average.of.Inverse.difference.normalized..INN.	61	0.5844	0.4241	0.7448	0.27
..8Average.of.Difference.variance	61	0.5833	0.4238	0.7428	0.277
..8Angular.Variance.of.Inverse.difference.normalized..INN.	61	0.5822	0.4255	0.739	0.283
..32Angular.Variance.of.Information.measure.of.correlation.1	61	0.5811	0.4318	0.7304	0.29
..8Average.of.Inverse.difference.normalized..INN.	61	0.58	0.4216	0.7384	0.297

..8Average.of.Dissimilarity	61	0.5789	0.4202	0.7376	0.304
..8Angular.Variance.of.Sum.variance	61	0.5778	0.4318	0.7238	0.311
..256Angular.Variance.of.Sum.entropy	61	0.5778	0.4226	0.733	0.311
..8Range.of.Difference.entropy	61	0.5767	0.4157	0.7377	0.318
..64Angular.Variance.of.Energy	61	0.5767	0.4290	0.7244	0.318
..8Average.of.Homogeneity	61	0.5756	0.4170	0.7341	0.325
..64Angular.Variance.of.Homogeneity	61	0.5756	0.4276	0.7235	0.325
..16Range.of.Entropy	61	0.5744	0.4186	0.7303	0.332
..8Range.of.Sum.variance	61	0.5733	0.4271	0.7195	0.339
..8Range.of.Homogeneity	61	0.5722	0.4155	0.729	0.347
Minimum	61	0.5711	0.4246	0.7176	0.352
..256Angular.Variance.of.Maximum.probability	61	0.5711	0.4227	0.7196	0.354
..8Angular.Variance.of.Entropy	61	0.5656	0.4085	0.7226	0.394
..32Angular.Variance.of.Difference.entropy	61	0.5656	0.4102	0.7209	0.394
..256Range.of.Energy	61	0.5656	0.4167	0.7144	0.394
..16Average.of.Entropy	61	0.5644	0.4145	0.7144	0.402
..256Range.of.Maximum.probability	61	0.5633	0.4144	0.7123	0.41
..8Average.of.Sum.entropy	61	0.5622	0.4164	0.7081	0.419
..16Average.of.Information.measure.of.correlation.1	61	0.5622	0.4113	0.7131	0.419
..16Angular.Variance.of.Energy	61	0.5622	0.4130	0.7115	0.419
..32Angular.Variance.of.Energy	61	0.5622	0.4131	0.7114	0.419
..8Average.of.Entropy	61	0.5611	0.4118	0.7104	0.427
..64Range.of.Sum.entropy	61	0.5611	0.4115	0.7107	0.427
..32Range.of.Sum.entropy	61	0.5589	0.4086	0.7091	0.444

..16Range.of.Information.measure.of.correlation.2	61	0.5578	0.4040	0.7116	0.453
..64Range.of.Energy	61	0.5578	0.4089	0.7067	0.453
..32Angular.Variance.of.Entropy	61	0.5567	0.4046	0.7087	0.462
..64Range.of.Difference.entropy	61	0.5567	0.4001	0.7132	0.462
..16Range.of.Homogeneity	61	0.5544	0.4011	0.7078	0.48
..32Range.of.Energy	61	0.5544	0.4044	0.7045	0.48
..8Angular.Variance.of.Energy	61	0.5533	0.4052	0.7015	0.489
..32Range.of.Correlation	61	0.5533	0.4013	0.7054	0.489
..8Range.of.Information.measure.of.correlation.2	61	0.5522	0.3991	0.7053	0.498
..16Angular.Variance.of.Information.measure.of.correlation.2	61	0.5522	0.3988	0.7057	0.498
..32Average.of.Entropy	61	0.5522	0.4025	0.702	0.498
..16Range.of.Correlation	61	0.5511	0.3992	0.703	0.507
..32Range.of.Information.measure.of.correlation.1	61	0.5511	0.4005	0.7017	0.507
..32Angular.Variance.of.Maximum.probability	61	0.5511	0.4008	0.7014	0.507
..64Range.of.Correlation	61	0.5511	0.3989	0.7033	0.507
..256Range.of.Correlation	61	0.5511	0.3989	0.7033	0.507
..8Angular.Variance.of.Homogeneity	61	0.55	0.3926	0.7074	0.517
..16Range.of.Energy	61	0.55	0.4003	0.6997	0.517
..32Angular.Variance.of.Sum.entropy	61	0.55	0.3992	0.7008	0.517
..64Range.of.Homogeneity	61	0.5489	0.4006	0.6972	0.526
..64Angular.Variance.of.Sum.entropy	61	0.5489	0.3980	0.6997	0.526
..16Range.of.Sum.entropy	61	0.5467	0.3958	0.6976	0.546
Kurtosis	61	0.5444	0.3979	0.691	0.565
..8Average.of.Information.measure.of.correlation.1	61	0.5444	0.3940	0.6949	0.565

..16Average.of.Energy	61	0.5444	0.3962	0.6927	0.565
..16Angular.Variance.of.Entropy	61	0.5433	0.3880	0.6986	0.575
..16Average.of.Sum.entropy	61	0.5422	0.3954	0.6891	0.585
..64Angular.Variance.of.Difference.entropy	61	0.5422	0.3878	0.6967	0.585
Standard.Deviation	61	0.5411	0.3845	0.6977	0.595
..8Angular.Variance.of.Information.measure.of.correlation.2	61	0.5411	0.3887	0.6935	0.595
..32Average.of.Energy	61	0.5411	0.3933	0.6889	0.595
..8Range.of.Energy	61	0.54	0.3916	0.6884	0.605
..16Average.of.Information.measure.of.correlation.2	61	0.54	0.3903	0.6897	0.605
..32Average.of.Information.measure.of.correlation.1	61	0.54	0.3873	0.6927	0.605
Percentile.99	61	0.5394	0.3863	0.6926	0.608
..64Average.of.Sum.entropy	61	0.5389	0.3913	0.6865	0.615
..8Average.of.Energy	61	0.5378	0.3895	0.6861	0.626
Maximum	61	0.5367	0.3819	0.6915	0.634
..16Average.of.Maximum.probability	61	0.5367	0.3895	0.6838	0.636
..32Average.of.Sum.entropy	61	0.5367	0.3895	0.6839	0.636
..32Angular.Variance.of.Correlation	61	0.5367	0.3841	0.6893	0.636
..64Average.of.Information.measure.of.correlation.2	61	0.5367	0.3870	0.6863	0.636
..16Angular.Variance.of.Homogeneity	61	0.5356	0.3831	0.6881	0.647
..256Angular.Variance.of.Difference.entropy	61	0.5356	0.3839	0.6872	0.647
..16Angular.Variance.of.Sum.entropy	61	0.5344	0.3841	0.6848	0.657
..64Angular.Variance.of.Correlation	61	0.5344	0.3819	0.687	0.657
..256Angular.Variance.of.Correlation	61	0.5344	0.3819	0.687	0.657
..16Angular.Variance.of.Correlation	61	0.5333	0.3808	0.6859	0.668

..32Average.of.Maximum.probability	61	0.5322	0.3842	0.6803	0.678
..32Range.of.Maximum.probability	61	0.5322	0.3815	0.683	0.678
..8Range.of.Correlation	61	0.5311	0.3787	0.6835	0.689
..16Range.of.Information.measure.of.correlation.1	61	0.53	0.3756	0.6844	0.7
..64Average.of.Information.measure.of.correlation.1	61	0.53	0.3794	0.6806	0.7
..64Average.of.Correlation	61	0.5289	0.3775	0.6803	0.711
..256Average.of.Correlation	61	0.5289	0.3775	0.6803	0.711
..32Range.of.Homogeneity	61	0.5278	0.3745	0.6811	0.721
..8Range.of.Maximum.probability	61	0.5267	0.3764	0.6769	0.732
..8Angular.Variance.of.Maximum.probability	61	0.5267	0.3764	0.677	0.732
..32Average.of.Correlation	61	0.5267	0.3754	0.6779	0.732
..64Average.of.Energy	61	0.5267	0.3727	0.6806	0.732
Percentile.1	61	0.5261	0.3734	0.6789	0.736
Percentile.95	61	0.5261	0.3721	0.6801	0.736
..8Average.of.Information.measure.of.correlation.2	61	0.5256	0.3766	0.6745	0.743
..16Angular.Variance.of.Maximum.probability	61	0.5256	0.3784	0.6727	0.743
..32Angular.Variance.of.Information.measure.of.correlation.2	61	0.5256	0.3734	0.6777	0.743
..8Range.of.Information.measure.of.correlation.1	61	0.5244	0.3688	0.6801	0.754
..64Range.of.Maximum.probability	61	0.5244	0.3727	0.6762	0.754
..32Average.of.Information.measure.of.correlation.2	61	0.5211	0.3689	0.6733	0.788
..64Angular.Variance.of.Sum.variance	61	0.5211	0.3729	0.6693	0.788
..16Average.of.Correlation	61	0.52	0.3694	0.6706	0.799
..64Range.of.Sum.variance	61	0.52	0.3721	0.6679	0.799
..8Angular.Variance.of.Correlation	61	0.5189	0.3663	0.6714	0.81

..256Range.of.Sum.variance	61	0.5189	0.3701	0.6677	0.81
..32Angular.Variance.of.Sum.variance	61	0.5178	0.3696	0.666	0.822
Percentile.5	61	0.5144	0.3607	0.6682	0.855
..8Average.of.Maximum.probability	61	0.5144	0.3677	0.6612	0.856
..32Range.of.Sum.variance	61	0.5144	0.3665	0.6624	0.856
..32Range.of.Information.measure.of.correlation.2	61	0.5122	0.3587	0.6657	0.879
..256Angular.Variance.of.Sum.variance	61	0.5122	0.3636	0.6608	0.879
..32Angular.Variance.of.Homogeneity	61	0.5111	0.3589	0.6634	0.89
..256Range.of.Difference.entropy	61	0.5111	0.3593	0.6629	0.89
..16Angular.Variance.of.Information.measure.of.correlation.1	61	0.5089	0.3556	0.6622	0.913
..256Average.of.Sum.entropy	61	0.5089	0.3579	0.6598	0.913
Mean	61	0.5078	0.3528	0.6628	0.925
..8Angular.Variance.of.Information.measure.of.correlation.1	61	0.5078	0.3540	0.6616	0.925
..64Average.of.Maximum.probability	61	0.5078	0.3554	0.6601	0.925
..16Range.of.Autocorrelation	61	0.5067	0.3552	0.6581	0.936
..32Range.of.Entropy	61	0.5067	0.3527	0.6607	0.936
..64Angular.Variance.of.Autocorrelation	61	0.5067	0.3569	0.6564	0.936
..16Range.of.Maximum.probability	61	0.5056	0.3575	0.6536	0.948
..32Angular.Variance.of.Autocorrelation	61	0.5044	0.3548	0.6541	0.959
..8Angular.Variance.of.Autocorrelation	61	0.5033	0.3540	0.6527	0.971
..16Angular.Variance.of.Sum.variance	61	0.5033	0.3551	0.6516	0.971
..64Range.of.Autocorrelation	61	0.5033	0.3526	0.654	0.971
..256Range.of.Autocorrelation	61	0.5033	0.3529	0.6537	0.971
..256Angular.Variance.of.Autocorrelation	61	0.5033	0.3534	0.6533	0.971

..64Average.of.Entropy	61	0.5022	0.3472	0.6572	0.983
..8Average.of.Correlation	61	0.5011	0.3512	0.6511	0.994
..16Range.of.Sum.variance	61	0.5011	0.3530	0.6492	0.994
..16Angular.Variance.of.Autocorrelation	61	0.5011	0.3511	0.6511	0.994
..32Range.of.Autocorrelation	61	0.5011	0.3503	0.652	0.994
..64Angular.Variance.of.Maximum.probability	61	0.5011	0.3484	0.6538	0.994
..8Range.of.Autocorrelation	61	0.5	0.3504	0.6496	1

The table in the search results presents the results of univariate analysis of the area under the receiver operating characteristic (ROC) curve (AUC) for various predictors in the context of endometrial carcinoma (EC) and malignant mixed Müllerian tumor (MMMT). The AUC is a measure of the performance of a classification model, with values ranging from 0 to 1. A higher AUC indicates better performance, with an AUC of 0.5 representing a random model.

The table is ordered by AUC and provides the following information for each predictor:

- Number of samples (N)
- AUC Lower limit (LL) of the 95% confidence interval
- Upper limit (UL) of the 95% confidence interval
- p-value

A predictor is considered significant if its AUC's lower limit (LL) is greater than 0.5. For example, the predictor "Volume" has an AUC of 0.7056, with a 95% confidence interval of 0.5692 to 0.8419 and a p-value of 0.00618, indicating that it is a significant predictor.

Some other significant predictors in the table include:

- 32Range of Cluster Shade (AUC: 0.6989, LL: 0.5576, UL: 0.8402, p-value: 0.00815)
- 256Angular Variance of Cluster Shade (AUC: 0.6989, LL: 0.5569, UL: 0.8409, p-value: 0.00815)
- 256Range of Cluster Shade (AUC: 0.6978, LL: 0.5559, UL: 0.8396, p-value: 0.00853)

Supplement Table S2. Clinical characteristics of the study patients. Patient demographics and clinical characteristics by group.

	EC (N=36)	MMMT (N=25)	Total (N=61)	p value
**Age**				**0.437**
Mean (SD)	64.25 (14.45)	66.52 (12.88)	65.18 (13.77)	
Median (Range)	67.00 (30.00, 94.00)	70.00 (24.00, 83.00)	68.00 (24.00, 94.00)	
**Race**				**0.026**
Asian	1 (2.9%)	2 (8.0%)	3 (5.0%)	
Black	3 (8.6%)	9 (36.0%)	12 (20.0%)	
Hispanic	9 (25.7%)	3 (12.0%)	12 (20.0%)	
White	22 (62.9%)	11 (44.0%)	33 (55.0%)	
**Pathology**				**< 0.001**
Endometrial carcinosarcoma	0	25		
Endometrial endometrioid adenocarcinoma, grade 1	5	0		
Endometrial endometrioid adenocarcinoma, grade 2	19	0		
Endometrial endometrioid adenocarcinoma, grade 3	2	0		
High grade endometrioid adenocarcinoma	4	0		
Endometrial endometrioid adenocarcinoma, poorly differentiated	1	0		

Other: clear cell, papillary, papillary serous, undifferentiated	5	0		
**FIGO Stage**				**0.047**
I	15	6		
II	5	5		
III	11	7		
IV	5	7		
**CA125**				0.109
≤ 35	14	10		
>35	9	4		
**Metastasis at presentation**				**<0.001**
No	30 (83.3%)	18 (72.0%)		
Yes	6 (16.7%)	7 (28.0%)		
**Distant metastasis location**				>0.54
Lung, Bone	1 (2.8%)	0 (0.0%)		
Malignant ascites & pleural effusions	0 (0.0%)	1 (4.0%)		
Omentum	1 (2.8%)	0 (0.0%)		
Omentum on path, peritoneal cytology	0 (0.0%)	1 (4.0%)		
Omentum, hepatic flexure, pleural effusion	0 (0.0%)	1 (4.0%)		

Paracolic gutter, omentum, + peritoneal cytology	0 (0.0%)	1 (4.0%)		
Peritoneal cytology, adrenal, lung	0 (0.0%)	1 (4.0%)		
Peritoneal carcinomatosis	3 (8.3%)	0 (0.0%)		
Peritoneal carcinomatosis, retroperitoneal	1 (2.8%)	0 (0.0%)		
Right chest wall	0 (0.0%)	1 (4.0%)		
Supraclavicular lymph node	0 (0.0%)	1 (4.0%)		
None	30 (83.3%)	18 (72.0%)		

Concise summary of the table results: **Age:** EC has a mean age of 64.25 years while MMMT has a mean of 66.52 years. The median ages are 67 and 70, respectively. The overall mean age for the entire sample is 65.18 years. Age differences are not statistically significant, with a p-value of 0.437. **Race:** Whites comprise the majority in both groups (62.9% in EC and 44% in MMMT). Black individuals are 8.6% in EC, but a notable 36% in MMMT. Racial differences are statistically significant, with a p-value of 0.026. **Pathology:** Only MMMT has cases of Endometrial carcinosarcoma (25 cases). Various subtypes of Endometrial endometrioid adenocarcinoma are present only in the EC group. The differences in pathology between the two groups are highly significant, with a p-value of <0.001. **FIGO Stage:** Both groups have varying numbers across stages I to IV, with the EC group predominantly in Stage I and the MMMT group dispersed more across the stages. There's a statistical difference in FIGO stages between the two groups with a p-value of 0.047. CA125: 14 EC patients and 10 MMMT patients have CA125 levels less than or equal to 35. 9 EC, and 4 MMMT patients have CA125 levels greater than 35. **Metastasis at Presentation:** Most (83.3%) of the EC group and 72% of the MMMT group did not have metastasis at presentation. However, metastasis was present in 16.7% of EC and 28% of MMMT patients. p-value: <0.001 (indicating significant difference in metastasis presence). **Distant Metastasis Location:** Various sites of metastasis are given for both groups. For instance, the EC group had 8.3% with peritoneal carcinomatosis, while MMMT had various sites, each with 4% occurrence, like malignant ascites & and pleural effusions, omentum on path, etc. **In conclusion, the two patient groups, EC and MMMT, showed significant differences in race, pathology, FIGO stage, and metastasis presentation but were similar in age, CA125 levels, and metastasis location.**

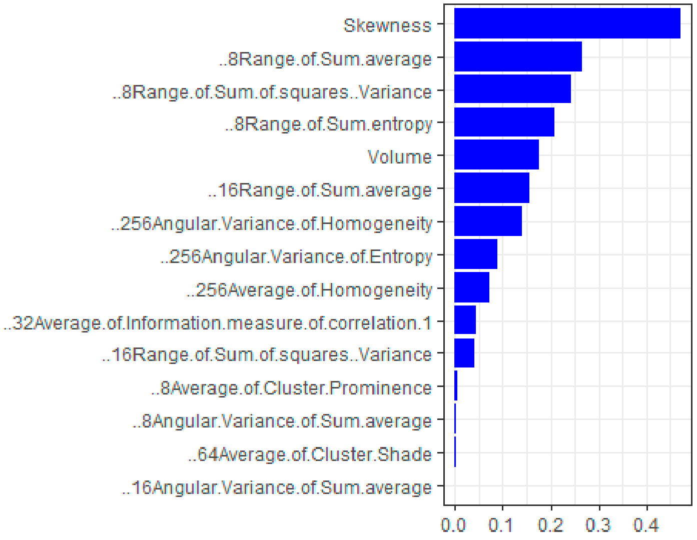

Supplement Figure S1. Final elastic net regularization model.

- 5-Fold Cross-Validation: The dataset is divided randomly into 5 folds. The model is trained and validated 5 times. In each iteration, the model is trained on 4 folds and validated on the remaining fold. This process is repeated until each fold serves as the test set once. This entire 5-fold cross-validation process is repeated 10 times to get more reliable results. The average Area Under the Curve (AUC) across all the validation runs is calculated as the performance metric for the model.
- Performance Metrics: The average AUC obtained from the 5-fold cross-validation is 0.685. The standard error associated with this average AUC is 0.0546. The AUC value indicates how well the model is able to distinguish between positive and negative instances. A higher AUC value generally indicates better discrimination.
- Predicted AUC using Entire Data: The model was trained on the entire dataset and the AUC obtained is 0.877. The range (0.791 to 0.962) indicates the confidence interval for this AUC estimate. The predicted AUC using the entire dataset is higher than the average AUC from cross-validation.
- Interpretation: The explanation points out that the predicted AUC using the entire dataset is likely an overestimate. This is because the model was trained and evaluated on the same data, potentially leading to overfitting. The suggestion is made that having an independent validation dataset, separate from the training data, would provide a more accurate evaluation of the model's performance. Independent validation helps assess how well the model generalizes to new, unseen data, reducing the risk of overfitting.
